# Morphological and molecular analysis of *Myxobolus malabaricus* n. sp. (Myxosporea: Myxozoa) parasitizing *Hoplias malabaricus* in Amazon basin

**DOI:** 10.1007/s11686-026-01354-6

**Published:** 2026-07-24

**Authors:** Márcia Nazaré Sacco Dos Santos, Sávio Lucas Matos Guerreiro, Flávia Letícia Gurjão De Andrade, Mikaela Vivian Pereira Andrade, Diehgo Tuloza Da Silva, Elane Guerreiro Giese, Edilson Rodrigues Matos, Igor Guerreiro Hamoy

**Affiliations:** 1 Animal Histology and Embryology Laboratory, Institute of Animal Health and Production, Federal Rural University of the Amazon, Belém, PA Brazil; 2 Laboratory of Human and Medical Genetics, Postgraduate Program in Genetics and Molecular Biology, Federal University of Pará, Belém, PA Brazil; 3 Laboratory of Applied Genetics, Federal Rural University of Amazonia, Socio- environmental and Hydrological Resources Institute, Belém, PA Brazil; 4 Carlos Azevedo Research Laboratory, Institute of Animal Health and Production, Federal Rural University of the Amazon, Belém, PA Brazil

**Keywords:** Parasite, Microscopy, Gill, Teleost fish, Cnidaria

## Abstract

The present study identified an undescribed myxozoan parasite species infecting *Hoplias malabaricus* specimens collected from the Arari River, in the municipality of Cachoeira do Arari in the state of Pará, northern Brazil. Thirty specimens were necropsied to search for microparasite, and when plasmodium found, material was collected for analysis via light microscopy, histology and molecular biology methodologies. Macroscopic plasmodium was observed in the gills of 30% of the examined specimens. Microscopic analysis of the plasmodium revealed the presence of myxospores belonging to the genus *Myxobolus*. These myxospores were distinguished from other *Myxobolus* species that parasitize freshwater Amazonian fish based on their overall morphology, morphometry and partial sequence (1,452 base pairs) of the SSU rDNA gene. These analyses revealed the existence of a new species of *Myxobolus*, named *Myxobolus malabaricus* n. sp. The new species is clearly distinct from other Myxozoa and may be an important parasite for causing disease in the gills of *H. malabaricus*, an important species of the Amazonian teleost fauna.

## Introduction

Myxozoans are obligate intracellular parasites of the phylum Cnidaria. They comprise more than 60 genera and thousands of species distributed across freshwater, marine, and brackish environments [1, 2]. The life cycle of these parasites depends on infecting both vertebrate and invertebrate hosts [3]. Studies on myxozoan have increased considerably in recent years, not only to the ecological and evolutionary importance of these parasites, but also to their impact on the health and commercial value of exploited fish species [4].

*Myxobolus* Bütschli, 1822, is a prominent myxozoan genus that includes numerous species infecting both wild and farmed fish [5, 3, 4]. Species of this genus are associated with several diseases, including lymphocytic meningoencephalomyelitis [6], branchial myxosporidiosis [7], myocarditis [8], and whirling disease [9]. In Brazil, *Myxobolus* species have been reported from different freshwater environments, with the Amazon region representing an important area for the diversity and discovery of this genus due to its high fish diversity and extensive aquatic ecosystems [8].

The estuary of the Amazon River forms a complex aquatic environment with high biological productivity that supports a substantial fish biomass, which is exploited by both artisanal and industrial fishing fleets. Within this estuary, Marajó Bay is one of the most important fishery zones for the local artisanal fleet and provides rich breeding and feeding grounds for many commercially important fish species [10, 11]. A number of myxozoan species, such as *Kudoa orbicularis* [12]. *Hoferellus azevedoi* [13] and *Ellipsomyxa arariensis* [14] have been described in fishes hosts around Marajó Island.

Among the important freshwater resources, the wolf fish *Hoplias malabaricus* Bloch, 1794, locally known as “traíra”, occurs in the vast majority of Central and South American river basins, and constitutes an important fishery and farmed resource in the Amazon region [15].

This knowledge gap has stimulated further parasitological research on *H. malabaricus*, collected from the central region of Marajó Island, which has resulted in the discovery of a new myxozoan species. Therefore, the aim of this study is to describe *Myxobolus malabaricus* n. sp., a new *Myxobolus* species, based on morphological traits and molecular phylogeny.

## Materials and methods

### Fish sampling

A total of 30 specimens of *H. malabaricus* were collected from the Arari River in the municipality of Cachoeira do Arari, located on Marajó Island, in the state of Pará, northern Brazil (1°10’03.3” S, 48°53’43.2” W), between May and October 2019. The specimens were transported alive (in aerated water from the natural environment) to the Carlos Azevedo Research Laboratory in the Institute of Animal Production and Health of the Federal Rural University of the Amazon (UFRA) in Belém, Brazil. The collection of specimens in the present study had been previously authorized by the UFRA Ethics Committee for Use of Animals in Research-CEUA (# 013/2014), and the capture of wild animals had been authorized through a license issued by the Brazilian Institute for the Environment and Renewable Natural Resources, IBAMA (SISBIO/ICMBio no. 27,119).

### Sample preparation, light microscopy and histological analysis

In the laboratory, the specimens were kept in aquaria, prior to being anaesthetized for necropsy using tricaine methane sulfonate (MS222, SIGMA) at a concentration of 50 mg.L^− 1^. The specimens were then examined under a stereomicroscope for detection of plasmodium microparasites on the surface of the body and in the internal organs. When microparasites were detected, they were collected and fixed for histological analysis, scanning electron microscopy (SEM) and molecular sequencing, using the appropriate fixer for each procedure. The spore morphometry was determined in accordance with Lom & Arthur, 1989, with mean measurements obtained from 30 fresh myxospores, all taken in the apical view. The fresh myxospores were photographed under a Zeiss Primo Star microscope equipped with an AxioCam Erc 5 camera and the images were processed in the AxioVision LE software.

For histological analysis, the parasites were fixed in Davidson’s solution (composed of 95% alcohol, formaldehyde, acetic acid and distilled water) for 24 h and then processed using the standard procedures for embedding in paraffin. Sections with a thickness of 5 μm were stained with hematoxylin and eosin (HE) and Ziehl-Neelsen [16].

For scanning electron microscopy (SEM), a plasmodium and myxospores were fixed in 5% glutaraldehyde buffered with sodium cacodylate (pH 7.2) for 12 h at 4 °C. They were then washed overnight in the same buffer solution and post-fixed in 2% OsO_4_ buffered with the same solution for 3 h at 4 °C. The samples were then dehydrated in an increasing series of ethanol concentrations. The plasmodium and myxospores were then dried to the critical point, metalized with a fine layer (20 nm) of gold, and photographed in a Hitachi TM 3000 [17].

### Molecular characterization and phylogenetic analyses

For the molecular analyses, myxospores were collected and fixed in 80% ethanol. DNA was extracted using the PureLink^®^ Genomic DNA mini extraction kit (Invitrogen, USA), following the “Mammalian Tissue and Mouse/Rat Tail Lysate” protocol supplied by the manufacturer. The DNA samples were quantified in a BioDrop Duo spectrophotometer (BioDrop, Cambridge, UK). The small subunit of the ribosomal DNA (SSU rDNA) was amplified using a polymerase chain reaction (PCR) with the universal eukaryote primers 18E (forward) [17] and 18R (reverse) [18], followed by a semi-nested PCR using the MX5/MX3 primer pair [19]. The PCRs were carried out in a final volume of 25 µL, containing 1 x Reddy Mix PCR master mix (Thermo Scientific, USA), 75 mM of Tris-HCl (pH 8.8), 20 mM of KCl, 0.1 (v/v) Nonidet P40, 1.5 mM of MgCl_2_, 0.2 mM of each nucleotide triphosphate (Thermo Scientific, USA), 10 pmol of each primer, 1.25 U of *Taq* DNA polymerase (Thermo Scientific, USA) and the DNA template (10–50 ng/µl).

The reaction conditions for the 18E/18R primers were 95 °C for 5 min, followed by 40 cycles of 95 °C for 1 min, 50 °C (annealing temperature) for 2 min and 72 °C for 4 min and 30 s, with a final extension at 72 °C for 10 min. The reaction conditions for the semi-nested PCR (primers MX5/MX3) were 95 °C for 5 min, followed by 35 cycles of 95 °C for 30 s, 56 °C for 30 s and 72 °C for 1 min, with a final extension at 72 °C for 10 min. The PCR products were confirmed by electrophoresis on 1% agarose gel, buffered with 1x Tris-Borate-EDTA (TBE) and stained with SYBR^®^ Safe (Invitrogen, USA) for visualization under blue light. The PCR products were purified using the ExoSAP-IT™ PCR product cleanup reagent (Thermo Fisher Scientific), according to the manufacturer’s instructions.

The PCR products were sequenced separately with the 18E/18R primers and then the MX3/MX5 primers. The sequencing reactions were performed using the Big Dye Terminator v3.1 cycle sequencing kit (Applied Biosystems, USA), following the manufacturer’s instructions, in an ABI 3100 genetic analyzer (Applied Biosystem). The sequences were aligned in the BioEdit software [20], and any ambiguous bases were resolved using the respective chromatograms. The sequences of the SSU rDNA gene sequences of myxozoan species deposited in GenBank were aligned in Clustal X 1.8 [21], at the standard settings, to determine the phylogenetic relationships among the species and the status of the new species.

The sequences used in the phylogenetic analyses were selected based on the highest similarity scores obtained using the Basic Local Alignment Search Tool (BLAST) [22]. *Kudoa alliaria* (DQ182561) was used as the outgroup in both phylogenetic analyses.

For the Bayesian inference analysis, the sequences were analyzed using MrBayes version 3.1.2 under the Markov chain Monte Carlo (MCMC) approach. Two simultaneous runs with four chains each were performed for 5,000,000 generations, with trees sampled every 500 generations [23].

The first 1,000 trees were discarded as burn-in, and posterior probabilities for each node were calculated from the remaining trees. Initial inspection of the trees was performed using TreeView X [24].

For the second phylogenetic reconstruction, sequences were aligned using the ClustalW algorithm [25] implemented in Geneious R11 (v11.1.5). A Maximum Likelihood (ML) phylogenetic analysis was performed using IQ-TREE v2.4.0, with the best-fitting nucleotide substitution model selected by ModelFinder according to the Akaike Information Criterion (AIC). The GTR + F+R6 model was selected as the optimal evolutionary model. Branch support was assessed using 10,000 ultrafast bootstrap replicates (UFBoot2). The tree was rooted using *Kudoa alliaria* (DQ182561) as the outgroup, and final tree visualization and graphical editing were performed using iTOL v1.6.12 [26].

Genetic distances were determined using PAUP 4.0b [27] with SSU rDNA gene sequences from a freshwater fish parasite, *Myxobolus* sp., which was grouped in the same clade as *Myxobolus malabaricus* n. sp.

## Results

**Fig. 1:**
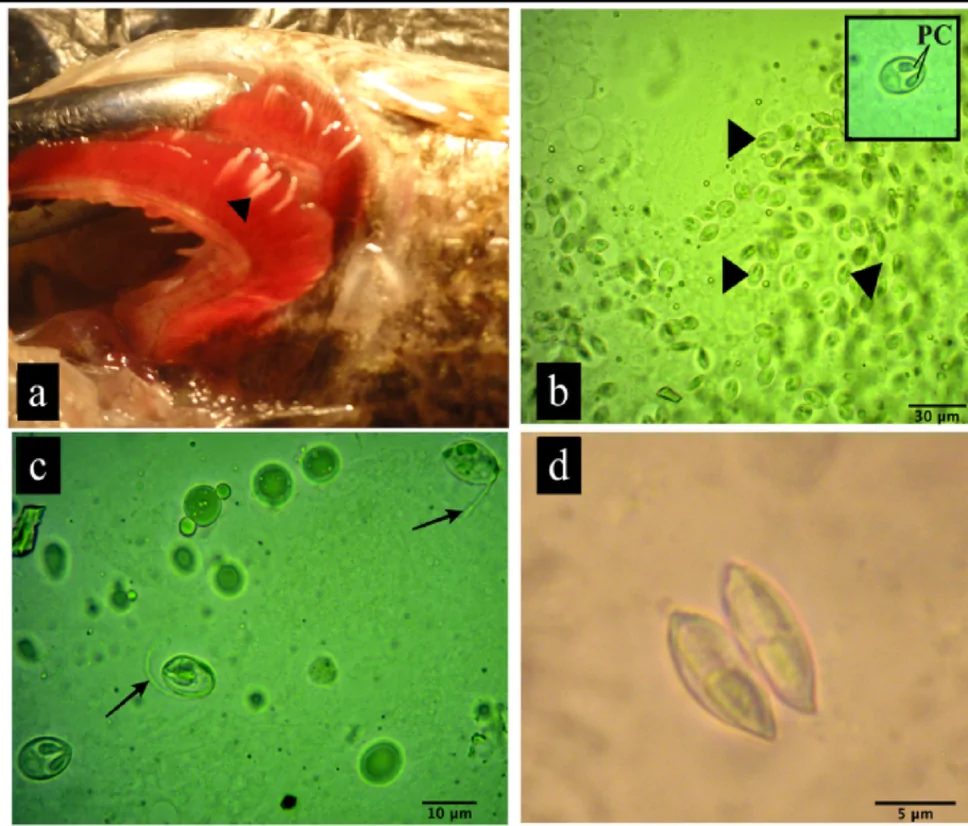
Plasmodium and myxospores of *M. malabaricus* n. sp.: **a** plasmodium observed in the branchial lamellae (arrowhead); **b** fresh myxospores of *M. malabaricus* n. sp. in frontal view (arrowheads), with the polar capsule indicated in the inset; **c** Myxospore of *M. malabaricus* n. sp. with an extruded polar tubule wrapped around the myxospore (arrow); **d** lateral view of fresh myxospores of *M. malabaricus* n. sp

Myxozoan infection was detected in 30% of the examined *Hoplias malabaricus*. Gross examination of the gills revealed the presence of whitish plasmodia embedded in the branchial tissue. Microscopic analysis showed mature myxospores displaying the diagnostic morphological characteristics of the genus *Myxobolus*, including ellipsoidal spores with two equal pyriform polar capsules positioned anteriorly (Fig. 1a–d). Histopathological examination of H&E-stained sections confirmed that the plasmodia were located within the branchial tissue (Fig. 4), providing additional evidence of the site of parasite development.

### Morphological description of ***M. malabaricus*** n. sp.

In the necropsy, whitish elongated plasmodia were observed macroscopically in the branchial lamellae of *H. malabaricus* (Fig. 1a). Microscopic examination revealed myxospores with morphological characteristics typical of the genus *Myxobolus.*

**Fig. 2:**
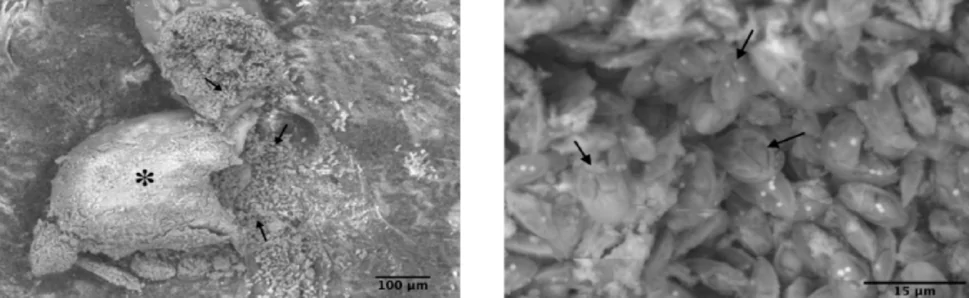
Plasmodium and myxospores de *M. malabaricus* n. sp. observed via scanning electron microscopy (SEM): (**a**) ruptured plasmodium of *M. malabaricus* n. sp. (*) with free myxospores (arrows); (**b**) myxospores of *M. malabaricus* n. sp. (arrows). Scale bars: (**a**) = 100 μm, (**b**) = 15 μm

#### Mature myxospore

**Fig. 3:**
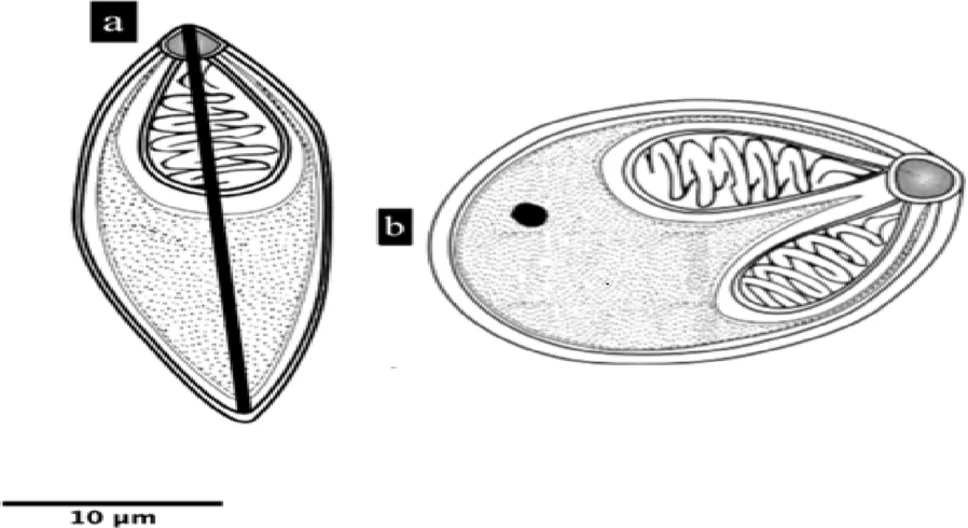
Schematic diagram of the myxospore of *M. malabaricus* n. sp.: (**a**) fresh spore in lateral view; (**b**) fresh myxospore in frontal view. Scale bar = 10 μm

The fresh myxospores were ellipsoid in shape, with two polar capsules of equal size and shape, arranged symmetrically in the apical region, parallel to the suture line (Figs. 1b and d and 3a). These details are consistent with those shown in Fig. 2, which used Scanning Eletronic Micrography.

The mature myxospores measured 10.2 ± 1.3 (8.9–11.5) µm in length and 6.19 ± 1.6 (4.6–7.8) µm in width, while the polar capsules were 3.78 ± 0.8 (3.0–4.6) µm long and 1.59 ± 0.6 (1.0–2.2) µm wide (Table 1). Each polar capsule had a polar tubule with 5–6 coils (Fig. 3).

**Table 1:** Comparison of myxospore morphometry among selected Myxobolus species infecting freshwater fishes, prioritizing species phylogenetically related to *M. malabaricus n.* sp. and species presenting similar myxospore morphology. SL, myxospore length; SW - myxospore width; PCL, polar capsule length; PCW - polar capsule width; NPT, number of polar tubule coils; GenBank accession number. All measurements are in µm

Species [Reference]	SL	SW	PCL	PCW	NPT	Genbank
*Myxobolus malabaricus* n. sp. [Present study]	10.2 ± 1.3	6.19 ± 1.6	3.78 ± 0.8	1.59 ± 0.6	5–6	OL334512
*Myxobolus macroplasmodialis* [28]	11 ± 0.5	8.5 ± 0.5	4.5 ± 0.5	2.8 ± 0.8	2	*KF296357*
*Myxobolus umidus* [29]	13.5 ± 0.7	7.8 ± 0.4	5.1 ± 0.4	2.7 ± 0.3	4–5	*KF296350*
*Myxobolus piraputangae* [29]	10.1 ± 0.5	8.7 ± 0.5	5.2 ± 0.4	3.0 ± 0.3	4–5	*KF296351*
*Myxobolus hilarii* [30]	11.5 ± 0.8	11.0 ± 0.7	6.5 ± 0.4	4.0 ± 0.2	5–7	*KM403404*
*Myxobolus aureus* [29]	12.6 ± 0.5	8.3 ± 0.3	5.7 ± 0.3	2.9 ± 0.2	7–8	*KF296348*
*Myxobolus batalhensis* [32]	15.2 ± 0.8	8.5 ± 0.5	5.2 ± 0.3	2.8 ± 0.2	6–9	*MF361090*
*Myxobolus axelrodi* [33]	20.5 ± 1.5	6.6 ± 0.9	9.9 ± 1.9	3.8 ± 0.6	-	*KU936090*
*Myxobolus tapajosi* [4]	15 ± 1.5	10.7 ± 1.1	5.8 ± 1.2	3 ± 0.7	6–7	MF193890

#### Histology

**Fig. 4:**
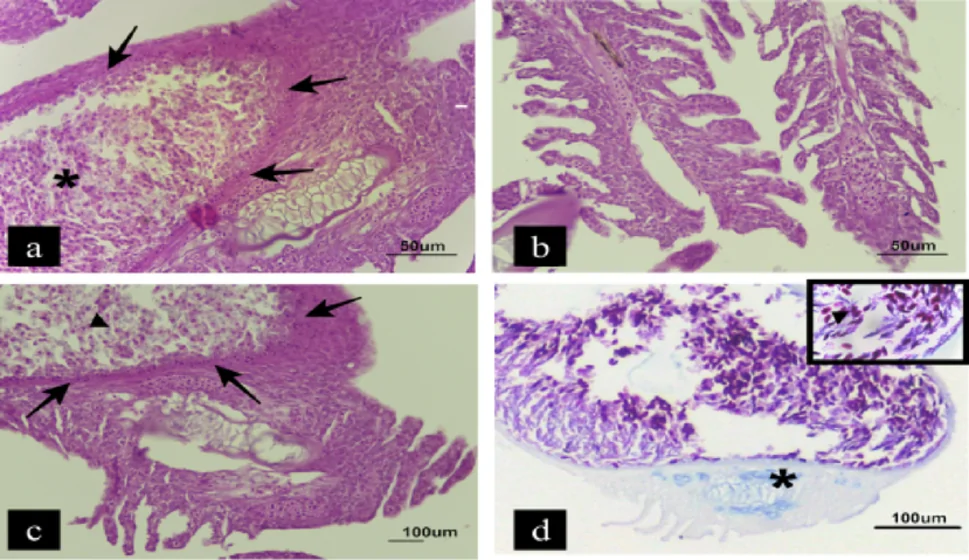
Gills of *H. malabaricus* infected with *M. malabaricus* n. sp.: **a** longitudinal section of a branchial lamella with presence of a plasmodium (*) enveloped in a connective tissue capsule (arrows); **b** longitudinal section of a branchial lamella; **c** detail of a plasmodium in a branchial lamella, containing mature and developing myxospores (arrowhead) and enveloped in a connective tissue capsule (arrows); **d** branchial lamellae (*). In inset, capsule of a stained with ZN (arrowhead), showing the myxospores that it contains

The histological analysis showed that the presence of regular cysts encapsulated by a connective tissue capsule and located in the primary lamellae of the host gill (Fig. 4a and c), causing a slight increase in the size of the gill filaments (Fig. 4b). Figure 4d shows myxospores of *M. malabaricus* n. sp. huddled together in the plasmodium. No inflammation was observed in the histological analysis.

### Taxonomic summary

Phylum Cnidaria Hatschek, 1888.

Subphylum Myxozoa (Grassé, 1970).

Class Myxosporea (Bütschli, 1881).

Order Bivalvulida (Schulman, 1959).

Family Myxobolidae (Thélohan, 1892).

Genus *Myxobolus* (Bütschli, 1881).

Species *Myxobolus malabaricus* n. sp.

Type host: *H. malabaricus* Bloch, 1794 (Characiformes: Erythrinidae).

Prevalence: 30%.

Infection site: Gills.

Etymology: The species epithet is derived from that of the host.

Locality: municipality of Cachoeira do Arari, Marajó Island, Pará state, Brazil.

Type material: A glass slide with a stained histological section of 5 μm thickness, containing the syntype of the new myxosporean species, was deposited in the Zoology Museum of the National Institute of.

Amazonian Research (INPA) in the municipality of Manaus, Amazonas state, Brazil, under the catalogue number 80/INPA.

Representative sequence: Partial SSU rDNA sequence, deposited in GenBank under accession number OL334512.

### Molecular and phylogenetic analyses

The partial sequence of the SSU rDNA gene sequence was obtained from the myxospore of *M. malabaricus* n. sp. Contains approximately 1,500bps (GenBank accession number OL334512). The Bayesian analysis showed grouping into two main clades, which were named A1 and A2, both containing myxobolid of several host families. Clade A1 was largely composed of *Myxobolus* species, while the majority of clade A2 is represented by the *Henneguya* species. *M. malabaricus* n. sp., which parasitize the gills of *H. malabaricus* (Characiformes), is the species that compound the clade A1, which is formed by *Myxobolus* that parasites the fishes of Characiformes order.

**Fig. 5:**
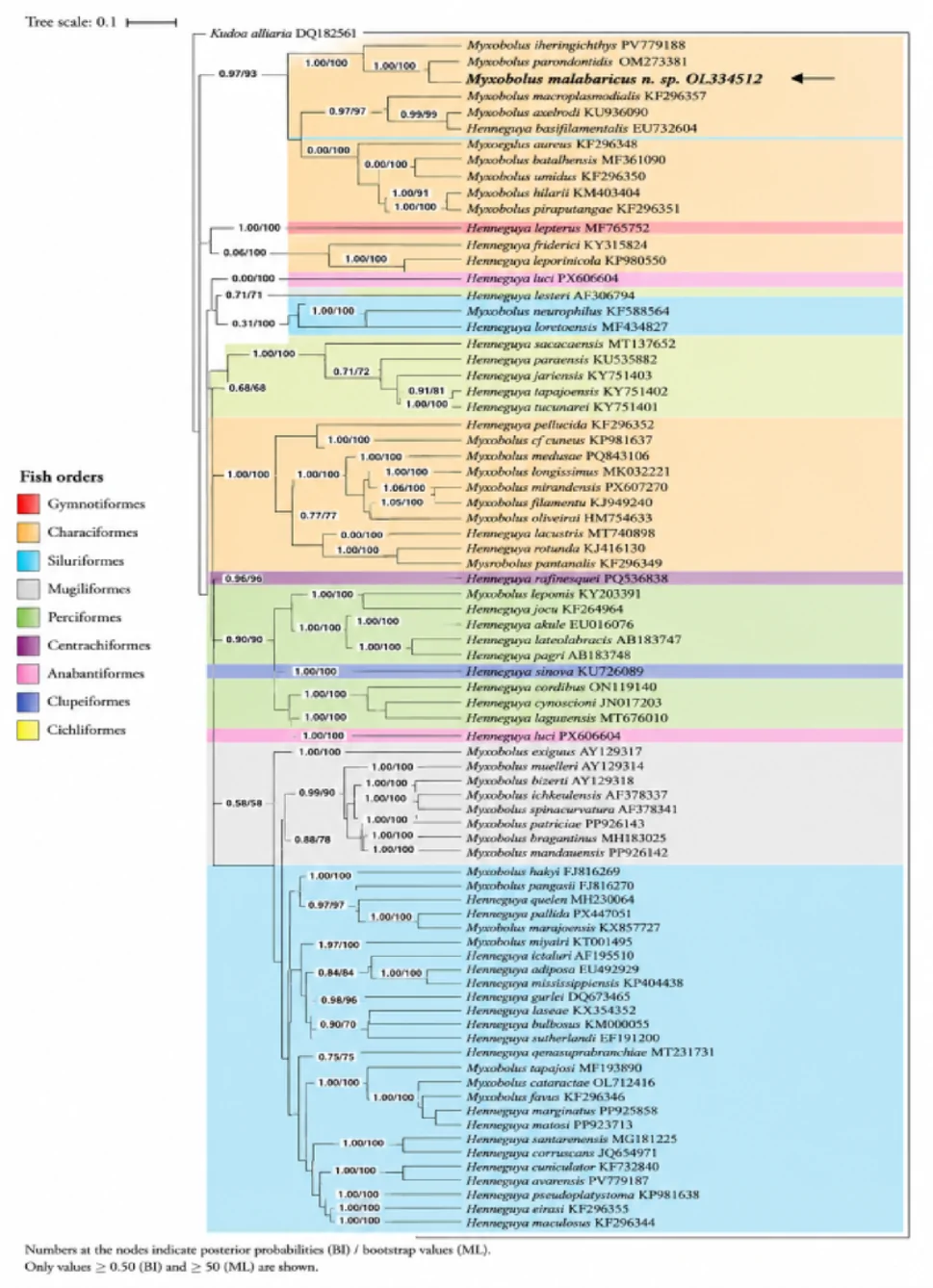
Phylogenetic relationships of *Myxobolus malabaricus* n. sp. infecting *Hoplias malabaricus* and selected myxozoan species inferred from partial SSU rDNA sequences using Maximum Likelihood (ML) and Bayesian Inference (BI). *Kudoa alliaria* was used as the outgroup. Numbers at the nodes represent Bayesian posterior probabilities (BI) and Maximum Likelihood bootstrap values (ML), respectively. Only BI values ≥ 0.50 and ML bootstrap values ≥ 50 are shown

Although *M. malabaricus* is similar to *Myxobolus macroplasmodialis* in the morphology and the morphometry, they are phylogenetically and geographically distinct (Fig. 5). This may be related to the fact that their hosts are from different regions of Brazil and the hosts are from different families.

The pairwise *p* distance between *M. malabaricus* n. sp. and other *Myxobolus* species that parasitize characiforms were all relatively high; among these distances, the shortest (18.55%) was recorded between *M. malabaricus* n. sp. and *M. aureus* (Table 2).

**Table 2:** Pairwise p-distance based on partial SSU rDNA sequences between Myxobolus malabaricus n. sp. and selected Myxobolus species infecting Neotropical freshwater fishes. GenBank accession numbers are provided after each species name

1. M. malabaricus *n*. sp. (OL334512)	1	2	3	4	5	6	7	8	9	10
2. *M. umidus* (KF296350)	0.2404									
3. *M. piraputangae* (KF296351)	0.2425	0.1244								
4. *M. hilarii* (KM403404)	0.2581	0.1272	0.0966							
5. *M. batalhensis* (MF361090)	0.2169	0.1636	0.1700	0.1690						
6. *M. aureus* (KF296348)	0.2157	0.1755	0.1819	0.1837	0.0842					
7. *H. basifilamentalis* (EU732604)	0.2518	0.2335	0.2252	0.2106	0.1827	0.1886				
8. *M. axelrodi* (KU936091)	0.2642	0.1859	0.2044	0.1754	0.1721	0.1760	0.1732			
9.*M. macroplasmodialis* (KF296357)	0.2291	0.2274	0.2185	0.2140	0.1853	0.2039	0.1901	0.1882		
10. *M. parodontidis* (OM273381)	0.2751	0.2711	0.2588	0.2450	0.2192	0.2193	0.2282	0.2180	0.2270	

## Discussion

The integrative approach employed in the present study, combining morphological, morphometric, histopathological, and molecular data, provides robust evidence supporting the establishment of Myxobolus malabaricus n. sp. as a new species parasitizing the gills of Hoplias malabaricus. Although myxospore morphology remains the basis for species descriptions within Myxobolus, numerous studies have demonstrated that morphological similarity alone is insufficient for reliable species delimitation because several species exhibit overlapping morphometric characteristics despite being genetically distinct [29, 30, 32, 37, 38]. Consequently, the integration of morphological and molecular evidence has become the current standard for myxozoan taxonomy [29, 44].

Morphologically, M. malabaricus n. sp. exhibits the diagnostic characteristics of the genus Myxobolus, including ellipsoidal myxospores with two equal polar capsules positioned anteriorly and symmetrically relative to the sutural line. However, species recognition within Myxobolus relies on the unique combination of diagnostic characters rather than on individual morphometric measurements [44]. In the present study, the new species can be distinguished from all congeners included in the comparative analysis by the combined characteristics of myxospore dimensions, polar capsule measurements, number of polar tubule coils, host species, infection site, and SSU rDNA sequence.

Among the species compared, Myxobolus macroplasmodialis is morphologically the most similar to M. malabaricus n. sp., mainly because both possess myxospores of comparable dimensions [28]. Nevertheless, these species differ in several stable taxonomic characters. Myxobolus macroplasmodialis parasitizes the abdominal cavity of Salminus brasiliensis, whereas M. malabaricus n. sp. develops in the branchial lamellae of H. malabaricus. Furthermore, differences in polar capsule dimensions and the number of polar tubule coils provide additional diagnostic characters separating the two species. Similar distinctions are also evident when M. malabaricus n. sp. is compared with M. piraputangae, M. umidus, M. aureus, M. hilarii, M. batalhensis, M. axelrodi, and M. tapajosi, all of which differ in one or more morphological or biological characteristics despite occasional overlap in spore size [4, 28, 29, 30, 32, 33].

The molecular analyses further reinforce the recognition of M. malabaricus n. sp. as a distinct species. The SSU rDNA gene has become the molecular marker most frequently employed in myxozoan taxonomy because it provides sufficient phylogenetic resolution while allowing comparisons among the majority of species currently deposited in GenBank [29, 44]. In the present study, both phylogenetic inference and genetic distance analyses demonstrated that M. malabaricus n. sp. constitutes an independent evolutionary lineage, despite clustering with other South American Myxobolus species infecting freshwater fishes. Similar phylogenetic approaches have successfully supported the description of several new species of Myxobolus parasitizing Neotropical fishes [29, 30, 32, 34, 37, 38].

The phylogenetic relationships recovered in the present study are consistent with previous investigations demonstrating that the evolution of myxozoans is influenced by both host phylogeny and tissue tropism [29, 40]. Species infecting phylogenetically related fish hosts frequently cluster together, particularly when they parasitize homologous organs, although this relationship is not absolute [29, 40, 41]. Therefore, the phylogenetic position of M. malabaricus n. sp. agrees with the current understanding that both host association and infection site contribute to the evolutionary diversification of Myxobolus species.

Histopathological analysis provided additional evidence regarding the interaction between M. malabaricus n. sp. and its host. The plasmodia developed within the primary branchial lamellae and were consistently surrounded by a well-defined connective tissue capsule. This encapsulation most likely represents a chronic host response that limits parasite expansion while preserving the structural integrity of the surrounding tissue. Similar connective tissue encapsulation has been reported in infections caused by other branchial myxozoans and is generally interpreted as an effective mechanism for containing parasite development without inducing severe inflammatory reactions [44, 45].

Interestingly, no inflammatory infiltrates were observed in association with the plasmodia. The absence of inflammation suggests that the host response to M. malabaricus n. sp. is predominantly chronic rather than acute. Similar observations have been reported for several species of Myxobolus and Henneguya, in which tissue damage is primarily associated with parasite growth and mechanical compression rather than inflammatory destruction [43, 44]. This pattern may reflect a relatively stable host-parasite relationship, allowing parasite development while minimizing extensive tissue injury.

Although inflammatory lesions were absent, the plasmodia promoted localized enlargement of the branchial lamellae through mechanical displacement of adjacent tissues. Because the gills perform essential physiological functions, including gas exchange, osmoregulation, acid-base regulation, and nitrogen excretion, structural alterations affecting the branchial architecture may compromise respiratory efficiency, particularly under conditions of high parasite intensity [43]. Similar pathological effects have been documented for other branchial myxozoans infecting freshwater teleosts, in which reductions in the functional respiratory surface result primarily from physical occupation of the branchial tissue rather than from inflammatory lesions [44, 45].

The relatively low pathogenicity observed in the present study should therefore be interpreted cautiously. Many myxozoan infections remain subclinical under natural conditions but may become clinically significant when fish are exposed to environmental stress, poor water quality, or intensive aquaculture conditions, where parasite transmission and infection intensity are generally increased [43, 45]. Consequently, although M. malabaricus n. sp. produced only moderate histopathological alterations in the examined specimens, its pathogenic potential under culture conditions deserves further investigation.

The Amazon Basin contains the greatest diversity of freshwater fishes worldwide and consequently represents one of the principal centers of myxozoan diversity. Nevertheless, only a small proportion of this parasite fauna has been formally characterized using integrative taxonomic approaches [4, 34]. The description of M. malabaricus n. sp. expands the known diversity of Myxobolus infecting Neotropical fishes and reinforces the remarkable diversity of myxozoans associated with Amazonian freshwater ecosystems.

Overall, the concordance between morphology, morphometry, host specificity, tissue tropism, histopathological characteristics, and molecular phylogenetic analyses provides consistent evidence supporting the recognition of Myxobolus malabaricus n. sp. as a valid new species. As demonstrated in recent studies describing South American myxozoans [29, 30, 32, 34, 37, 38], the integration of multiple sources of evidence has become essential for accurately documenting the diversity and evolutionary relationships of Myxozoa.

## Conclusions

The integrative morphological and molecular analyses (partial SSU rDNA) support the description of *Myxobolus malabaricus* n. sp. as a new species parasitizing the gills of *Hoplias malabaricus*. This study expands the knowledge of the diversity of *Myxobolus* species infecting Amazonian freshwater fishes and contributes to the understanding of myxozoan biodiversity in the Amazon Basin.

## Data Availability

Representative sequence: 18E/18R+MX5/MX3, deposited in GenBank under accession number OL334512; A glass slide with a stained histological section of 5 μm thickness, containing the syntype of the new myxosporean species, was deposited in the Zoology Museum of the National Institute ofAmazonian Research (INPA) in the municipality of Manaus, Amazonas state, Brazil, under the catalogue number 80/INPA. The data generated during the study are included in this article.
